# A Novel Hybrid Secure Image Encryption Based on Julia Set of Fractals and 3D Lorenz Chaotic Map

**DOI:** 10.3390/e22030274

**Published:** 2020-02-28

**Authors:** Fawad Masood, Jawad Ahmad, Syed Aziz Shah, Sajjad Shaukat Jamal, Iqtadar Hussain

**Affiliations:** 1 Department of Electrical Engineering, Institute of Space Technology, Islamabad Highway 1, Islamabad 44000, Pakistan; Fawadkttk@gmail.com; 2 School of Computing, Edinburgh Napier University, Edinburgh EH10 5DT, UK; J.Ahmad@napier.ac.uk; 3 School of Computing and Mathematics, Manchester Metropolitan University, Manchester M15 6BH, UK; 4 Department of Mathematics, College of Science, King Khalid University, Abha 62529, Saudi Arabia; shussain@kku.edu.sa; 5 Department of Mathematics, Statistics, Physics, Qatar University, Doha 2713, Qatar; iqtadarqau@qu.edu.qa

**Keywords:** chaotic maps, confusion, diffusion, fractals, Lorenz chaotic maps, shuffling, non-linearity, hybrid dynamical system

## Abstract

Chaos-based encryption schemes have attracted many researchers around the world in the digital image security domain. Digital images can be secured using existing chaotic maps, multiple chaotic maps, and several other hybrid dynamic systems that enhance the non-linearity of digital images. The combined property of confusion and diffusion was introduced by Claude Shannon which can be employed for digital image security. In this paper, we proposed a novel system that is computationally less expensive and provided a higher level of security. The system is based on a shuffling process with fractals key along with three-dimensional Lorenz chaotic map. The shuffling process added the confusion property and the pixels of the standard image is shuffled. Three-dimensional Lorenz chaotic map is used for a diffusion process which distorted all pixels of the image. In the statistical security test, means square error (MSE) evaluated error value was greater than the average value of 10000 for all standard images. The value of peak signal to noise (PSNR) was 7.69(dB) for the test image. Moreover, the calculated correlation coefficient values for each direction of the encrypted images was less than zero with a number of pixel change rate (NPCR) higher than 99%. During the security test, the entropy values were more than 7.9 for each grey channel which is almost equal to the ideal value of 8 for an 8-bit system. Numerous security tests and low computational complexity tests validate the security, robustness, and real-time implementation of the presented scheme.

## 1. Introduction

The application of multimedia information communication has grown dramatically in our daily lives. The multimedia data transmission necessitates high transmission rates and protection. The medical imaging systems, military image databases, and pay-per-view TV are such applications where preservation plays a fundamental role in the requirement of a multimedia system.

With the passage of time, we are increasingly encountering various kinds of vulnerabilities and security loopholes in wired and wireless communication media such as Wi-Fi, Ethernet, and so on. The digital world has changed the lives of human beings comparing it to earlier decades. The analogue transformation to digital bitstream was one of the groundbreaking discoveries of the well-known scientist Claude Shannon in the year 1949, the same person introduced the property of confusion and diffusion. The property of randomness helped in securing digital multimedia information. The security of digital bitstream became one of the central issues when the data was transformed into a digital bitstream. The privacy of digital information posed a new problem in the digital world. The secure digital data in the form of binary bits are openly accessible for hackers. The data can be easily obtainable from the far site using the internet. It needs proper measurements to secure digital information over an insecure line of communication [1,2,3]. The digital data can be secured by hiding the identity of the original digital image over the secret cover image or another approach is pixels distortion or encryption. The first method of securing digital information is information hiding techniques and the study of steganography, which means the value of the data is preserved under the secret image. The image security is a very important issue as compared to textual security, where image pixels are to be examined concerning nearby pixels in a different orientation. The more pixels are dissimilar means the proposed encryption technique is more suitable for brute force attacks. Moreover, plain image pixels are always correlated to each other where an attacker can easily find secret information. Chaos-based encryption technique is preferred over some other existing methods because of less computational power, fast, and accurate. The future work is also extended to the direction of quantum-based cryptography, chaos quantum-based cryptography, and post-quantum random bit generators [4,5,6,7,8,9,10,11,12].

In the last few years, a large number of encryption algorithms schemes were proposed to secure the digital information from eavesdroppers and advertisers such as international data encryption algorithm (IDEA), data encryption standard (DES), and advanced encryption standard (AES) [13,14,15,16,17,18,19,20,21,22]. The foremost importance is given in implementing resolute protection while studying the hybrid properties of confusion and diffusion which was proposed by Claude Shannon as in [23,24]. Due to some of the major concerns including large data capacity, low entropy value and high correlation in the existing algorithms make it inappropriate for image encryption. The chaos-based encryption is a fast-growing area for image encryption. The use of chaotic maps, multiple chaotic maps, and hybrid dynamical systems of chaos-based encryption has attracted a large number of researchers and nowadays, the majority of scholars, researchers, and engineers are proposing security schemes based upon chaotic cryptography [25]. The highly pseudorandom sequences generated through chaotic maps provide strong entropy and least correlation between pixels of the ciphered image. Chaos-based encryption has brought the stretched gap between the plain and ciphered image which makes it challenging for invaders and attackers to break the code due to its robustness of the key generated through chaotic maps. Chaos-based encryption has certain unique properties which makes it superior over other approaches for encryption of plain digital images [11,26,27,28,29].

In addition, fractals are geometric shapes having non-linearity on all scales. It possesses randomness which makes it suitable for implementing secure and reliable cryptosystems. The system can be generated using the complex values of fractals in a complex domain. The chaotic nature of fractals using a private and public key is capable of securing digital multimedia information. The sensitivities and dependence on initial conditions produces complexities for an intruder to create the key to decipher the confidential information. The fractals generate infinitely complex patterns and show repetition and self-similarity at different scales. It is the repeating process of images and is familiar patterns since the whole universe consists of fractals in different forms [30,31,32,33,34,35,36,37,38,39,40,41,42,43]. In this context, we have designed a cryptosystem based on shuffling with Julia set of fractals key and chaos theory. The proposed algorithm at the stage of fractals generates random complex numbers for image encryptions. The complex numbers necessitate the extraction of real numbers to encrypt the plain image pixels. This system was secured with shuffling, but the histograms in the statistical section showed that the system can further be enhanced by the addition of chaotic maps providing the hybrid system. The fractals’ random numbers are treated with the three-dimensional Lorenz chaotic map to gain robust security and validate all the security tests. The pair of histograms are shown in the subsequent section of the statistical tests. The statistical tests validated the proposed system. 

The rest of the paper is composed as follows. Section 2 demonstrates the nonlinear mechanism and properties of the chaotic maps. In Section 3, the literature review of the work is addressed. Section 4 incorporates the methodology of the presented scheme. Section 5 elaborates the steps needed to develop a secure scheme. Section 6 will show the assessed results of the utilized secure algorithm and its comparison with already existing schemes. Section 7 covers the software and system specification needed to design the secure system. Finally, Section 8 is a brief discussion and conclusion of the paper by briefing the findings of the suggested cryptosystem.

## 2. Nonlinear Mechanism 

**Figure 1:**
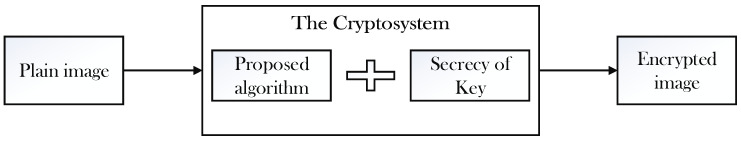
Basic schematic chart of image encryption.

A nonlinear process is a simple non-linear difference equation emerged in different fields of science—for instance in biology, physics, engineering, economics, and social sciences—which possess different dynamic behaviors which are pertinent to chaos or cryptography [44,45,46,47,48]. Chaos cryptography or chaotic systems have some properties for instance randomness nature, sensitive to the initial condition, aperiodic, and ergodicity which makes it unique for designing a secure cryptosystem. If the initial condition value is insignificantly changed the output at other ends will show immensely fickleness. Chaos behavior exists surrounding when looking into nature [49,50,51,52,53,54,55]. The basic schematic chart of image encryption is shown in Figure 1.

### Properties of Chaotic Maps

1. Deterministic: The chaotic maps are the deterministic dynamical non-linear systems. This means that if we recognize the initial condition, then the system can be determined easily else the system will behave chaotic unstable system [56].
2. Sensitivity to initial condition: The chaotic maps are highly sensitive to the starting condition of the system. The wrong keys will neither encrypt the image correctly nor decrypt the image properly. The change in the key will show highly strange attractors [56].
3. Randomness: The system will generate pseudorandom sequences. The sequences generated through chaos-based will be highly complex to be determined and prognosticated [30].
4. Unstable: The system will be unstable in the chaotic region. The Lyapunov exponent will determine the actual chaotic and non-chaotic regions [30].
5. Ergodicity of the chaotic maps: The encryption algorithm performance will have the same distribution for any plain text [30].

## 3. Literature Review 

This section presented a literature review based on the existing algorithms for digital image encryption schemes. The basic operation is XOR which is used to encrypt the digital multimedia images for secure information. The XOR operation is bit by bit operation between binary numbers. The image can be decrypted by taking the XOR operation again. This is a very simple method to distort the pixels of the plain images. 

In [57], the authors developed a scheme of encryption for digital multimedia information by experimental comparison of the chaotic and non-chaotic map. The cryptosystem used the discrete cosine transform, followed by Bernoulli map and permutation of pixels. The correlation coefficient test, entropy, quality of encryption, avalanche effect, number of pixels changing rate unified average changing intensity, and key sensitivity test approved the proposed system. In [30], Masood et al. proposed a system based on Mandelbrot fractals and Fibonacci series followed by piecewise chaotic Kaplan–Yorke map. The hybrid system utilizing the Mandelbrot set of fractals generated the complex values. The real values are extracted from complex values and are treated with a chaotic map. The proposed system is investigated using different security tests for its validation. The system passed all the security tests and validated it for real-time communication. In [31], Agarwal introduced a system based on Mandelbrot fractals. The key generated by Mandelbrot fractals is further treated with discrete two-dimensional chaotic sequences. The system is followed by shuffling and complex *XOR* operation. The system is studied using specific statistical tests that authorized the suggested system. In [58], Batool et al. proposed a hybrid system based on image shuffling followed by pixel distortion. The pixels are initially shuffled using Arnold cat map for the channels of Lena and pepper as standard test images. The scrambled image is subjected to an encrypted phase where the pixels are distorted channel-wise. The Lucas sequence encrypted all the channels. The proposed system was validated using extensive experiments. Kumar et al. in [59] introduced a technicality entirely based on four-dimensional Lorenz chaotic map. The authors generated a random number matrix to improve security by using the hyperchaotic system. This method encrypted five different types of grey channel images including, the Mona Lisa test image, black, cameraman, vegetable, and rice. The proposed technique was passed through various tests to evaluate the validity of the system. The aforementioned encryption schemes can be applied on a number of different applications discussed in Refs [60,61,62,63,64].

## 4. Proposed Technique for Secure Cryptosystem

The main objective of the work is to determine a secure cryptosystem. In this communication, we initiated the designation of the system by the generation of fractals at a different value of C and then utilized chaotic based three-dimension Lorenz system. The algorithm is investigated several times for different test images. The results of various statistical tests for different images are shown in the subsequent section of statistical parameters. The proposed system is valid for real time communication.

### 4.1. Initial Shuffling Process

The grey channels red, green, blue (R, G, B) are initially randomly permuted to achieve the property of confusion. The shuffling process helped us obtaining partial security. however, the shape of the histograms of the shuffled grey channels looks same as the plain grey channels (R, G, B) and are shown in the subsequent section of statistical tests. 

### 4.2. Julia Set of Fractals 

Julia’s work is associated with a complex plane and the unique points for which the series generated through the $Z_{n+1}=Z_{n}^{2}+C$ does not go to the infinity. The C in the Julia set indicates the complex constant. The Julia fractals set changes with the change in the complex constant value C. The value must be smaller to generate Julia’s set of fractals. The value of (C<1) generates the desired quadratic based fractals. The different values of C depicts different shapes of fractals [65]. The equation of quadratic Julia is the conformal mapping so in the case of conformal the angles are preserved. Suppose ‘J’ be the Julia set then $x^{\prime }\to x$ leaves J invariant. 

The quadratic Julia set of the system can be illustrated as

(1) $$f(z)=z^{2}+c$$

For almost every value of ‘C’ will generate different types of fractals. The system behaves like chaos dynamical system by setting the value exact to C = −0.745429, and $C_{x}=0,$ $C_{y}=0$. 

The Julia set capacity dimension can be illustrated as

(2) $$d_{capacity}=\frac{1+|c{|}^{2}}{4\ln2}+O(|c{|}^{3})$$

**Figure 2:**
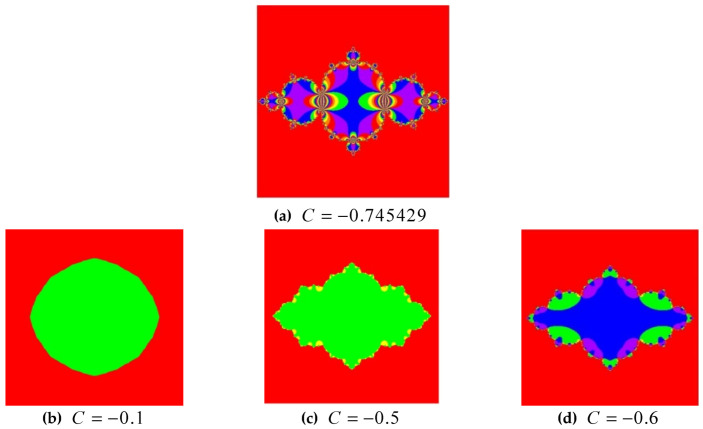
Generated shapes (**a**–**d**) at (*C* < 1).

Different shapes of fractals are generated using Julia set of fractals with varying the value of C. The fractals give no shape when the value of (C>1). The four distinct shapes of fractals are shown for the case of when (C<1) as shown in Figure 2. 

The complex shape of fractals is generated when the value of C is set to be = −0.745429. From Figure 2, it is clear that the proposed cryptosystem is secure.

### 4.3. Three-Dimension Chaotic Lorenz Map

The Lorenz is a three-dimension chaotic dynamical map. The combined differential equation was developed by one of the notable scientist Edward Lorenz in 1963 [66]. The attractor generated through the Lorenz chaos sequences is the deck of chaotic solution for the Lorenz system. The plotting of the Lorenz system generates the attractor which looks like Butterfly. The chaotic system was initially developed for atmospheric convection. The system can be described using the simple formula

(3) $$\begin{array}{l} \frac{dx}{dt}=\sigma (y-x),\\ \frac{dy}{dt}=rx-rz-y,\\ \frac{dz}{dt}=xy-bz. \end{array}$$

**Figure 3:**
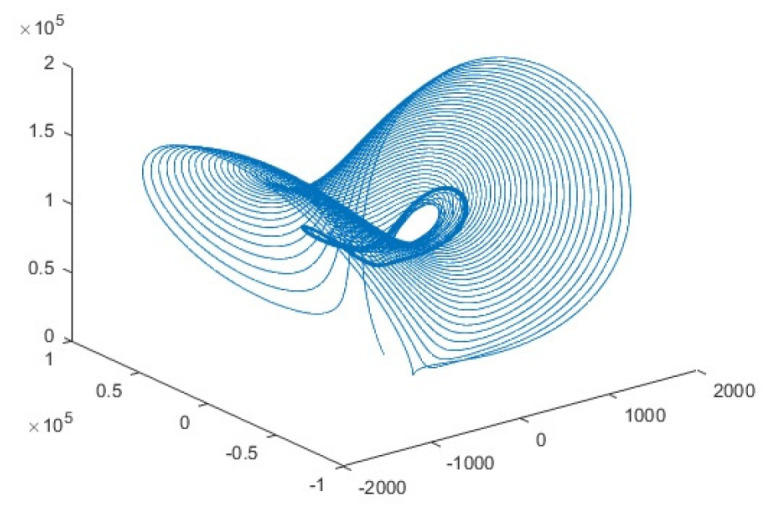
3D Lorenz chaotic attractor.

The system majorly depends upon the control parameters, *r*, and *b*. The system produces chaos sequences when fixing the precise value of chaos. The trajectory of the system is achievable utilizing the Runga Kutta algorithm. The system presented chaos behavior and encrypt the channels for the rho (Rayleigh number) = 88500, (Sigma) = 10, and b (Beta) = 8/3. The system exhibited more excellent performance encrypting the channel wise images and appended the additional layer of security over the layer of fractal-based encrypted channels. The security of channels is reviewed here the advantage of the Lorenz chaotic map and following the Lorenz chaotic map. The addition of Lorenz’s chaotic map added much more randomness than fractals key-based encryption. The attractor that is generated by utilizing three dimensional Lorenz chaotic map is shown in Figure 3. 

## 5. A New Cryptosystem Based on Fractal Function and 3D Lorenz Chaotic Map

### 5.1. The Encryption Process

1. The test image splash having the size of 512 × 512 × 3 is used for encryption on the suggested cryptosystem.
2. Convert the plaintext test image into three individual grey channels of red, green, and blue possessing the same size of 512 × 512.
3. Shuffle previously divided channel pixels to achieve partial security.
4. Produce the complex values from the complex domain of the Julia set of fractals.
5. Deduce the real values from the Julia set of fractals produced in step 4.
6. The real values of Julia’s set of fractals are multiplied with the shuffled pixels in step 3.
7. Design three dimensional Lorenz chaotic map and bitwise XOR with output random stream of values that are generated in step 6.
8. Collect three highly random encrypted channels having a size of 512 × 512. Combine the three encrypted channels utilizing the cat command to produce a colored image having a size of 512 × 512 × 3.

### 5.2. The Decryption Process

**Figure 4:**
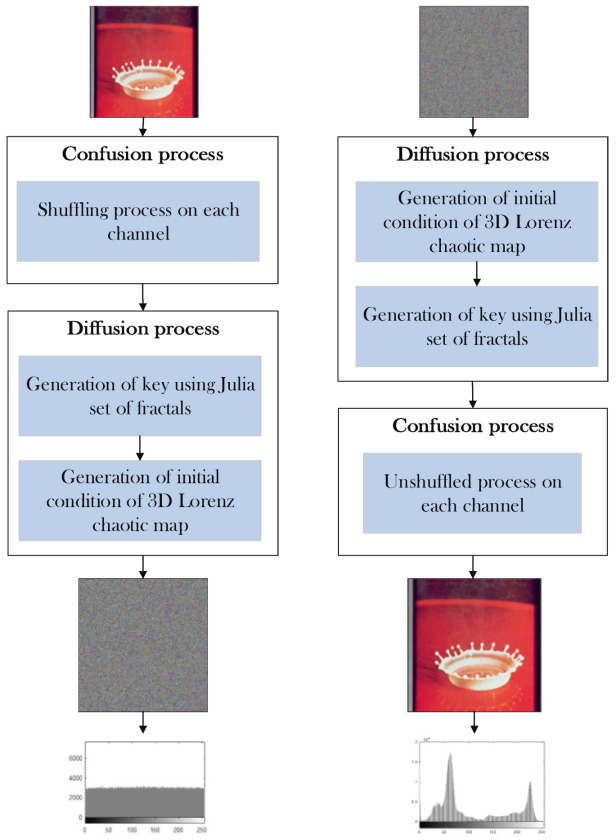
Encryption and decryption process.

**Figure 5:**
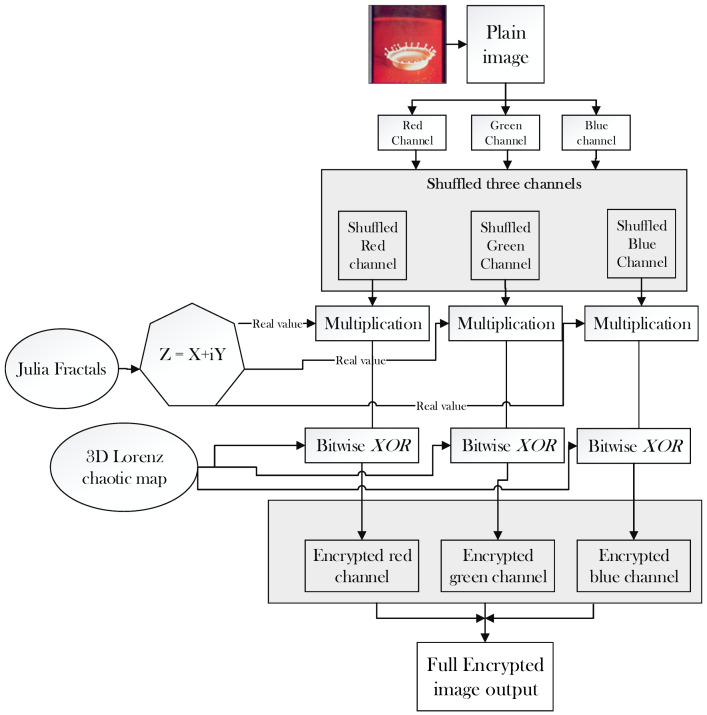
Flow chart for the proposed algorithm.

9. The colored encrypted image possessing a size of 512 × 512 × 3 is classified into three grey layers of encrypted channels (R, G, B) having a size of 512 × 512 sequentially.
10. Each channel is transferred through inverse by exerting bitwise XOR again for the three-dimensional Lorenz chaotic map to get the stream of values produced by Julia set of fractals.
11. The random values generated in step 6 of the encryption stage is classified by the Julia set of fractals.
12. The real values are now combined with the imaginary value to get the complex values of fractals.
13. In this step, the pixels are unshuffled to get into the respective grey channels with same size.
14. The grey channels having a size of 512 × 512 is combined using the cat command to get 512 × 512 × 3 full layered color image of a splash. The process of encryption and decryption and flow chart of the complete process is shown in the below Figure 4 and Figure 5 respectively.

## 6. Security Evaluation of Proposed Scheme

The section comprises statistical tests that are implemented to the suggested hybrid system. The secure scheme is produced using a channel-wise shuffling process and is inserted to fractal function to produce the random bits. The random bitstream is then treated with the chaos-based 3D Lorenz dynamical systems. Some of the statistical tests are cumulated by using plain and encrypted images. The major tests include mean square error (MSE), peak to signal noise ratio (PSNR), mean absolute error (MAE), randomness test, number of pixels changing rate (NPCR), unified average changing intensity (UACI), and computational time. The following tests validated the proposed scheme. The analysis and security tests of pixels are performed in this section as shown subsequently in subsections. 

**Figure 6:**
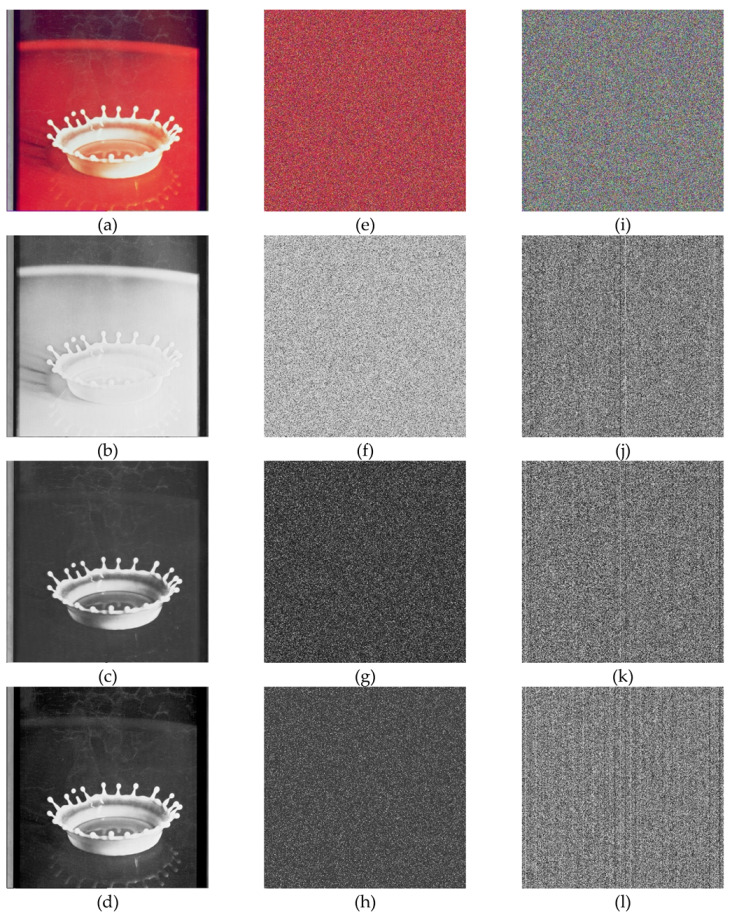
Plain and Encrypted images of splash (512 × 512); (**a**–**d**) Plain image of splash and respected three channels of a splash test image. (**e**–**h**) Shuffled image of splash and respected three channels of splash image. (**i**–**l**) Encrypted image of splash and respected three channels of splash test images using Julia fractals.

**Figure 7:**
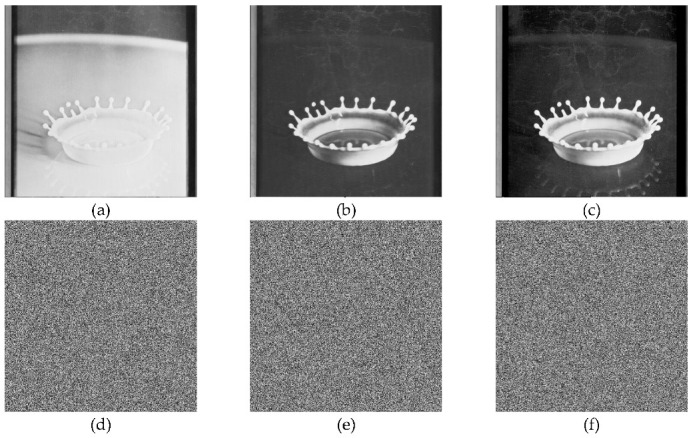
Plain and Encrypted images of splash (512 × 512); (**a**–**c**) Three respected plain channels of a splash test image. (**d**–**f**) The final encrypted channels using 3D Lorenz chaotic maps.

**Figure 8:**
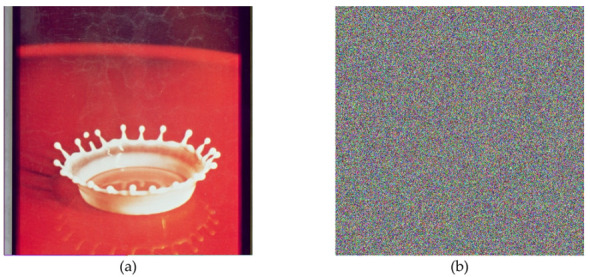
Plain and encrypted image of splash (512 × 512); (**a**) The plain image of a splash. (**b**) The final encrypted image of splash image using 3D Lorenz chaotic map.

**Figure 9:**
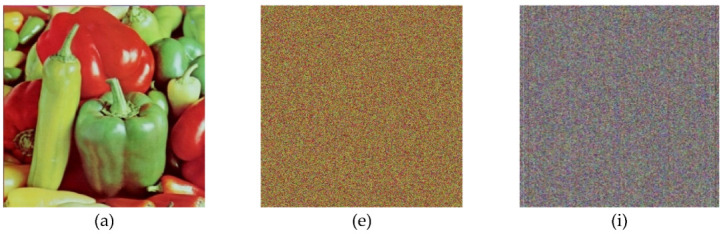

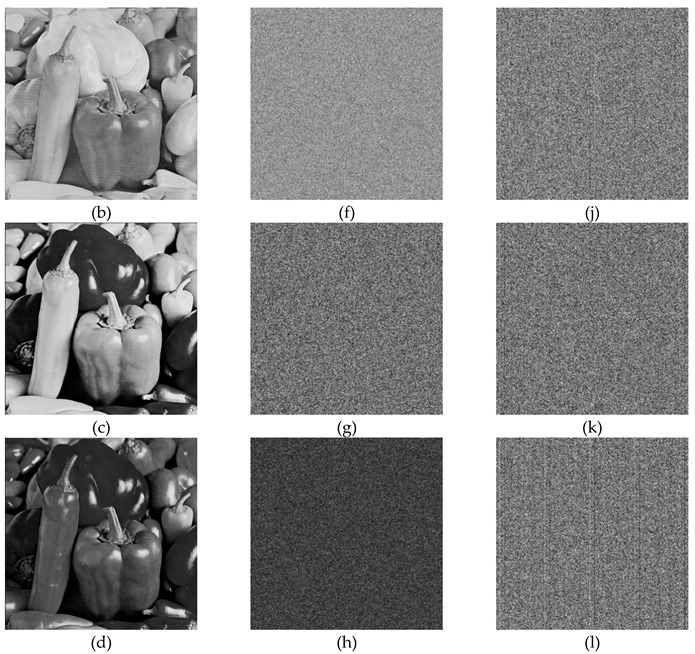
Plain and encrypted images of pepper (512 × 512); (**a**–**d**) Plain image of pepper and respected three channels of a pepper test image. (**e**–**h**) Shuffled image of pepper and respected three channels of pepper image. (**i**–**l**) Encrypted image of pepper and respected three channels of pepper test image using Julia fractals.

**Figure 10:**
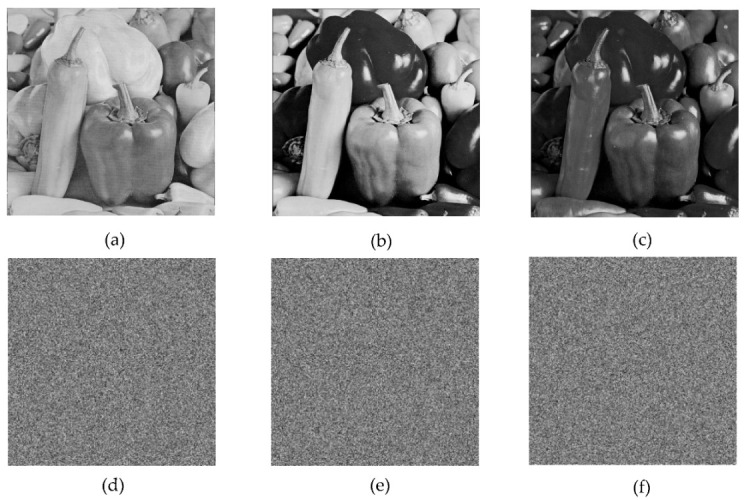
Plain and encrypted images of pepper (512 × 512); (**a**–**c**) Three respected plain channels of a pepper test image. (**d**–**f**) The final encrypted channels using 3D Lorenz chaotic maps.

**Figure 11:**
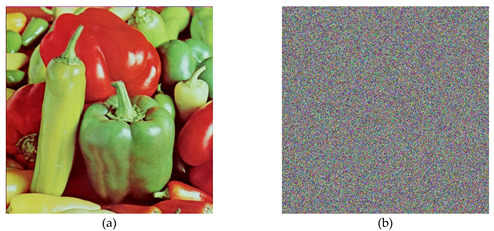
Plain and Encrypted image of pepper (512 × 512); (**a**) The plain image of a pepper. (**b**) The final encrypted image of the pepper image using 3D Lorenz chaotic map.

**Figure 12:**
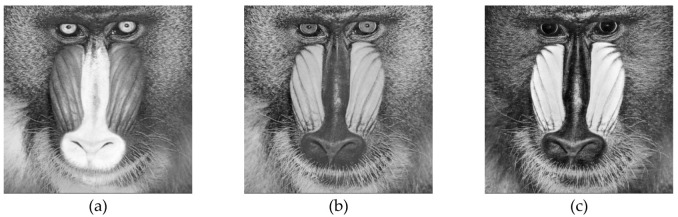
Plain channel-wise test images of the baboon (512 × 512); (**a**–**c**) Three respected plain channels of a baboon test image.

**Figure 13:**
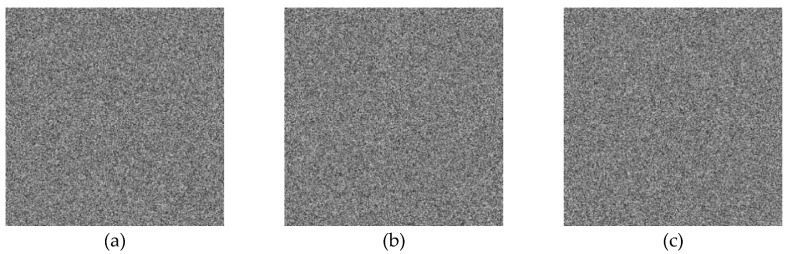
Encrypted channel-wise test images of the baboon (512 × 512); (**a**–**c**) Three respected encrypted channels of a baboon test image using 3D Lorenz chaotic map.

**Figure 14:**
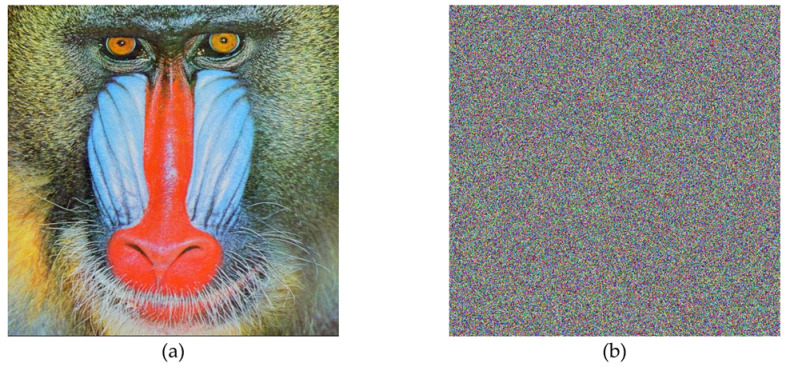
Plain and encrypted image of the baboon (512 × 512); (**a**) The plain image of a baboon. (**b**) The final encrypted image of the baboon image using 3D Lorenz chaotic map.

In the below Figure 6a–d are the plain images of a splash having the original size of 512 × 512; while Figure 6e–h are the layer-wise splash images that are shuffled using Arnold map. The third column of Figure 6i–l are encrypted three layers of splash image which shows partial security during the process of image encryption using Julia set of fractals. In the subsequent Figure 7a–c are the plain images of splash while the partially secured channels are subjected to three dimensional Lorenz chaotic maps that improved the security level of the proposed scheme. Figure 7d–f are the fully secured encrypted three channels. Finally, full colored plain and encrypted image having a size of 512 × 512 × 3 are shown in Figure 8a,b. The proposed algorithm is utilized on several other images as well. In the following Figure 9a–d are the plain image of pepper with its three layers; while Figure 9e–h are the shuffled full colored pepper image and its three layers. The encrypted three channels of pepper image which represents partial security during the process of image encryption using Julia set of fractals are shown in Figure 9i–l. The image is subjected to three dimensional Lorenz chaotic map. The high level of security is achieved after the additional layer of using Lorenz chaotic maps. In Figure 10a–c and Figure 10d–f are the final plain and encrypted channels of pepper image. The full-dimensional colored plain and encrypted pepper image having a size of 512 × 512 × 3 is shown in Figure 11a,b respectively. The Figure 12a–c are three plain layers of baboon images as shown; while the fully encrypted layers of baboon image of red, green and blue are shown in Figure 13a–c. The full colored plain and encrypted image of baboon having a size of 512 × 512 × 3 are shown in Figure 14a,b.

### 6.1. Histogram Analysis

**Figure 15:**
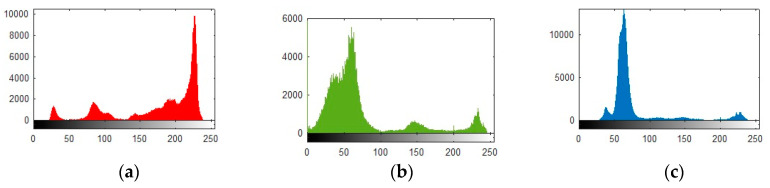

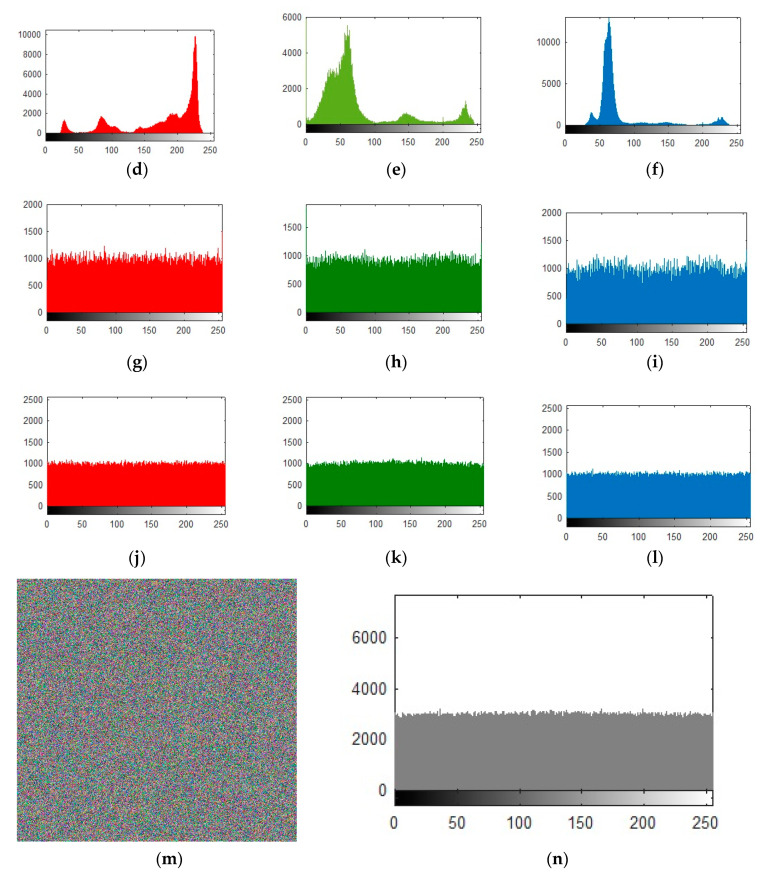
Plain and encrypted histogram of splash image (512 × 512); (**a**–**c**) Plain histograms for three grey channels of splash image. (**d**–**f**) Shuffled histograms for three grey channels of splash image. (**g**–**i**) Encrypted three channels of splash image using Julia fractals. (**j**–**l**) Final histograms of three channels using 3D Lorenz chaotic maps. (**m**,**n**) Final histogram of combined three channels for the splash test image.

**Figure 16:**
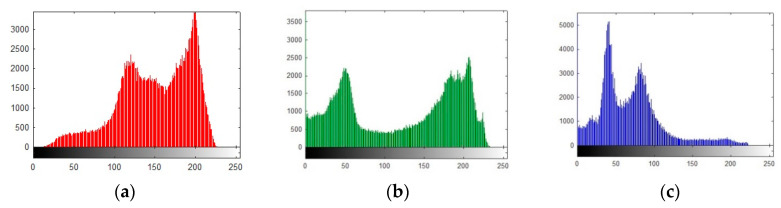

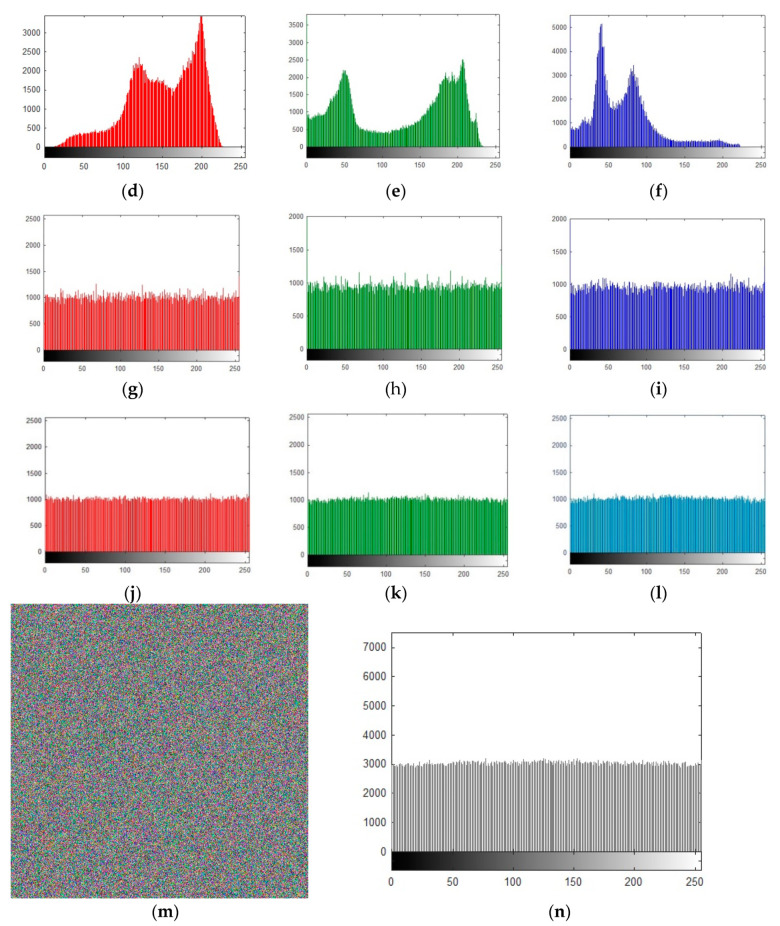
Plain and encrypted histogram of pepper image (512 × 512); (**a**–**c**) Plain histograms for three grey channels of pepper image. (**d**–**f**) Shuffled histograms for three grey channels of pepper image. (**g**–**i**) Encrypted three channels of pepper image using Julia fractals. (**j**–**l**) Final histograms of three channels using 3D Lorenz chaotic maps. (**m**,**n**) Final histogram of combined three channels for the pepper test image.

The histogram analysis is one of the most popular test that is used to estimate the robustness of the proposed cryptographic algorithm. The strength of the proposed scheme can be calculated by the distribution of pixels in its range. The minima and maxima range falls in 0–255 for 8-bit images. The up and down pixels in the plain image shows that the advertiser can attack the vulnerable bumpy pixels to guess the exact location of confidential information. The uniformity of pixels elaborates that confidential information is highly secured from any type of attack, thus the intruder is incapable of differentiating the pixels or guessing the quantity of information. It is essential to have uniform pixels for encrypted images. The up and down pixels show that the pixels did not achieve maximum randomness. The up and down pixels with minimum randomness reveals that the data is easily accessable. In Figure 15a–f the histograms of the three channels e.g., red, green, and blue depicts that the pixels information is insecure. The up and down pixels are breakable. In Figure 15g–l are the histograms of all the three respected channels of splash image are uniform that shows that the pixel information is secure and is not easily breakable. Finally, the whole colored plain and encrypted image having size of 512 × 512 × 3 are shown Figure 15m,n. The test is also applied on pepper image having the same size of 512 × 512 × 3. In Figure 16a–f are the histogram of three channels e.g., red, green, and blue. The non-uniform distribution of pixels reveals that the digital information is insecure for any type of communication. In Figure 16g–l are the histograms of secured channel when the images are subjected to secure cryptosystem. The Figure 16m,n is fully secured pepper image histogram for the combined three layers of colored image. The above information of histogram analysis demonstrate that the high level of security is achieved when the system is subjected to extra layer of chaotic map based on 3D lorenz system. The final channels wise histogram pixels in Figure 16j–l are smoothy distributed with no bumpy area. The above statements validated the suggested scheme.

### 6.2. Correlation Analysis for the Adjacent Pixels

**Figure 17:**
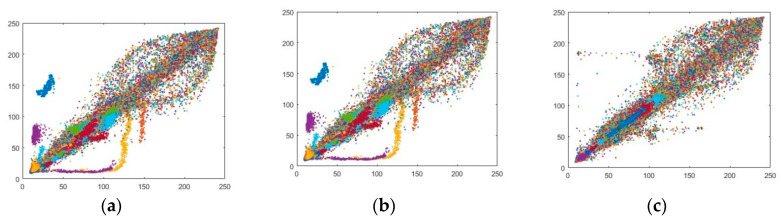

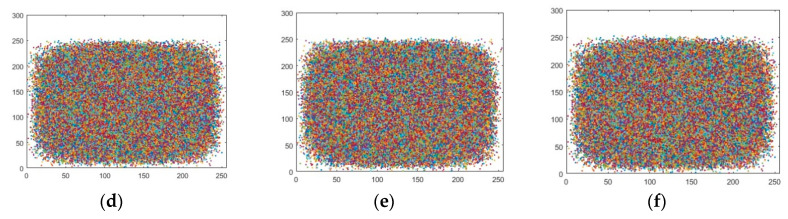
Correlation coefficient of splash image (512 × 512); (**a**–**c**) Correlation coefficient of plain splash image for three directions of a = Horizontal (H-D), b = Diagonal (D-D) and, c = Vertical (V-D). (**d**–**f**) Correlation coefficient of encrypted splash image for three directions of d = Horizontal (H-D), e = Diagonal (D-D) and f = Diagonal (V-D).

**Figure 18:**
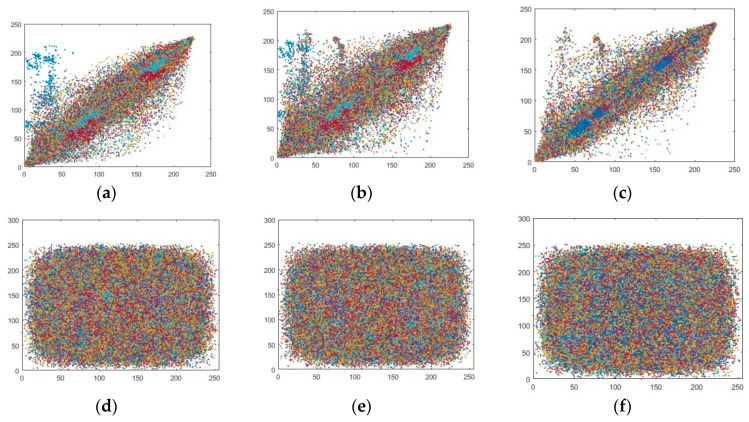
Correlation coefficient of pepper image (512 × 512); (**a**–**c**) The correlation coefficient of plain pepper image for three directions of a = Horizontal (H-D), b = Diagonal (D-D) and, c = Vertical (V-D). (**d**–**f**) The correlation coefficient of encrypted pepper image for three directions of d = Horizontal (H-D), e = Diagonal (D-D) and f = Vertical (V-D).

The adjacent pixel analysis is also known as a correlation coffecient test which is one of the momentous tests to gauge the quality of encryption by relating the pair of variables. In this case, the pair of variables are plain text and ciphered text. The correlation coefficients take the value between two extreme points of [+1, −1]. The value that is nearing 1 explicates that the two-variable pixels are extremely dependent and there is an excellent correlation that survives between plain and ciphertext while the value of −1 shows the pair of variables are different from one another which explicates that there are highly dissimilarity exists. The value of 0 means that there is no relation between the two variables. In the case of perfect correlation, the two images are the same while in the case of getting the value of −1 reveals that the two images are distinct. The value must be small enough to gain satisfying security of the image. Mathematically, the correlation coefficient can be depicted as:

(4) $$r=\frac{\mathrm{cov}(x,y)}{\sigma_{X}\times \sigma_{y}},$$

where $\sigma_{X}=\sqrt{\mathrm{var}(x)}$ and $\sigma_{y}=\sqrt{\mathrm{var}(y)}$.

(5) $$\mathrm{var}(x)=\frac{1}{N}\sum_{i=1}^{N}{\left(x_{i}-E(x)\right)}^{2},$$

(6) $$\mathrm{cov}(x,y)=\frac{1}{N}\sum_{i=1}^{N}\left(x_{i}-E(x)\right)(y_{i}-E(y)),$$

where *x* and *y* in the aforementioned equations are plain image pixels and encrypted image pixels whereas, *M* × *N* is the total dimension of the image. Figure 17 and Figure 18 show the pixel distributions along with three directions of horizontal direction (H-D), vertical direction (V-D), and diagonal direction (D-D) pixels. The a–c in Figure 17 and Figure 18 are the pixel distribution along three directions of splash and pepper images which are in the form of text possessing a dimension 512 × 512 × 3. The pixels forming the diagonal lines depicts that the pixels are correlated to each other. The excessive amount of pixel’s similarity is always in the high risk. Whereas in the case of Figure 17 and Figure 18d–f the pixels are interspersed in the range of 0–256. The distribution of pixels covering the whole range shows that the values are highly dissimilar from each other which reveals that the cryptographic system is secure against attack. 

Table 1 designates adjacent pixels values for three different directions. The system is investigated for six different test images possessing a dimension of 512 × 512 × 3. The values of the plain image of splash for three different directions of horizontal, vertical, and diagonal are 0.9839, 0.9773, and 0.9913 which is imminent to the value of 1. The proposed splash image is encrypted and tested its pixels values dissimilarities along horizontal, vertical and diagonal directions are 0.0011, 0.0037, 0.0029 which is approaching 0 exhibits that the pixels are highly dissimilar from each other. Any type of attack is not possible on high non-correlated encrypted images. 

Table 2 shows the comparison of the pixel’s values in three different directions. The standard image of splash has corresponded to certain existing cryptosystems. The tests authorized the new hybrid based designed system and guaranteed that the designed cryptosystem is very strong compared to previously designed systems. 

### 6.3. Mean Absolute Error

This is one of the widely use standard analysis that is used to investigate the robustness of the proposed system. The value must be greater to validate the proposed scheme. The M × N is the cumulative dimension of the standard image. The $P_{i,j}$ is the plain image and $E_{i,j}$ is the encrypted image. The system can be illustrated as

(7) $$MAE=\frac{1}{M\times N}\sum_{i=0}^{M-1}\sum_{j=0}^{N-1}\left|E_{i,j}-P_{i,j}\right|$$

Table 3 consists of six standard test images. The analysis is applied to the encrypted images of the proposed system. It is important to accomplish a larger value to pass the test which confirms the robustness of the proposed cryptosystem. The standard test images of MAE values are displayed in the subsequent Table 3. The average value should be in the range of 65 to 70. The reliability of the system entirely depends on the greater value of MAE. The greater values depict that the attained cryptographic system has better resistivity against the differential attacks. Different values are calculated in the subsequent Table 3 for different standard images. The dimension of 512 × 512 for each test image is kept constant. The values are compared to already existing cryptosystems. The aforementioned information authenticated the proposed system.

### 6.4. Differential Attack Analysis

Two types of tests are employed to attain sensitivity or differential attack analysis. The number of pixels changing rate (NPCR) and unified average changing intensity (UACI). These tests are used against differential attacks. The tests signify the chance of occurrence of the attack and its sensitivity towards the source image by changing the value. The tests are elaborated in the following subsection.

#### 6.4.1. Number of Pixel Changing Rate

The NPCR or number of pixel changing rate manifests the possibility of the differential attack by its sensitivity. The highly sensitivity of the system shows that the generated algorithm is sturdy against any probable attacks. The tests can be estimated by taking two encrypted images and one plain image. The variation in the encrypted images will occur with the change in the respected plain image. This shows that any petite change in the plain image will give an entirely different encrypted image. In simple words, it illustrates the percentage of the different pixels of encrypted images at the same position whose plain image is edited for the single pixel. The system can be calculated in percentage. The ideal value of NPCR is perpetually 100. The value approaching 100 shows that the proposed system is robust against any differential attack.

Let $E_{1}$ and $E_{2}$ be the two encrypted images whose source plain image is differed by a single pixel. The system is illustrated as

(8) $$NPCR=\frac{\sum_{i,j}F(i,j)}{W\times H}\times 100\%$$

where $F_{i,j}$ = 0 for $E_{1(i,j)}=E_{2(i,j)}$, and $F_{i,j}=1$ for $E_{1(i,j)}\neq E_{2(i,j)}$.

Whereas *W* × *H* is the width and height (total size) of the image. In Table 4, the values of NPCR are calculated layer-wise for different standard test images having a size of 512 × 512. The computed values of NPCR ≥ 99.60. The value of splash red channel is = 99.62, the value of green channel is = 99.61 and the value of blue channel is = 99.62 with an average = 99.62. The results demonstrate that the system is near to the ideal value which is 100. The system has guaranteed that the designed system is applicable for real-time communication. 

#### 6.4.2. Unified Average Changing Intensity 

Unified average changing intensity (UACI) is one of the important analysis of sensitivity tests. It is mandatory to have robust security. The test is based upon the intensity difference between two images. The system can be computed using the equation

(9) $$UACI=\frac{1}{W\times H}\sum_{i,j}\left[\frac{E_{1(i,j)}-E_{2(i,j)}}{255}\right]\times 100\%$$

whereas the *W* × *H* is the cumulative size of the standard image. $E_{1}$ and $E_{2}$ are two encrypted images at *i*th row and *j*th column. The test is applied to three different encrypted images having a size of 512 × 512 channel-wise. The average value of UACI is 33 while the computed UACI values of standard splash image value is 33.90, Tiffany obtained value is 36.26 and, airplane is equal to 32.50 as shown in Table 4.

In Table 5, the proposed encrypted image UACI value is compared to several existing secure system values. The proposed value of UACI is superior over the existing cryptosystems. The test ensured the proposed system values are highly satisfying the security criteria. The proposed system is valid for any type of secure communication. 

### 6.5. Mean Square Error

It is important to have accuracy in the proposed system. The system without accuracy confronts different types of external attacks. The mean square error (MSE) test is used to find the accuracy of the suggested system by using the plain and encrypted images of the proposed system. The system can be computed as

(10) $$MSE=\frac{1}{M\times N}\sum_{i=1}^{M}\sum_{j=1}^{N}\left(O_{\left(i,j\right)}-E_{\left(i,j\right)}\right)$$

Mean square error (MSE) must be greater in value to resist differential attack. The greater value of MSE shows that the proposed system is robust against any type of attack. The cryptographic algorithm has been investigated on various standard images to confirm the validity of the proposed scheme. In the subsequent paragragh, the calculated values are tabulated for six different standard images including the proposed image of splash.

In Table 6 The MSE values for the standard proposed test image of splash is investigated. The computed red layer is value is 11412.96, the value of green layer is 12272.97 and the value of blue layer is 9908.84. The secure system is investigated on several other test images. The average value of 11198.25 for the splash image is much greater than the average value of 10000. The average value of the pepper is 10842.43, the computed value of the baboon is 10905.36, the tiffany image value is equal to 12743.12, the evalauted fruit image value is 10034.06, and the airplane image is 10347.71. The result indicates that the proposed system is checked layer-wise and in the combined state. 

In Table 7 the proposed image also compared to various exisiting schemes e.g., AES, AES-CBC, AES-Counter, AES-Feedback, AES-Stream.

### 6.6. Peak to Signal Noise Ratio 

Peak to signal noise ratio is an important analysis for the suggested system to evaluate the quality of the image. The system can be illustrated as

(11) $$PSNR=10\log_{2}\frac{I_{\max}^{2}}{MSE}$$

The value of mean square error (MSE) and peak to signal noise ratio (PSNR) is always inversely to each other. The ample value of MSE with its lower value of PNSR signifies good security. The values of a peak to signal noise ratio (PSNR) for the splash image for a red layer is 7.59, green layer is 7.28, and a blue layer is 8.20 as shown in Table 6. 

The results are tabulated in Table 6 and Table 8. The values of PSNR is evaluated for all the three channels for certain standard images is shown in Table 6. In Table 8 the average values are tabulated for mean square error (MSE) and peak to signal noise ratio (PSNR); the average calculated value of splash image is 7.69, following the average evaluated value of pepper image is 7.81, the value of baboon image is 7.79, similarily the tiffany is 7.38, the fruit image is equal to 8.16, and finally 8.08 is calculated value of airplane standard test image. 

The results in Table 6 and Table 8 indicates that the proposed system values are remarkable compared to already systems developed to date. The system has ensured the strongness against brute force attacks.

### 6.7. Entropy 

Information entropy is a powerful analysis used to find the unpredictability and randomness in the suggested scheme. The important term was originally used by the notable scientist, Claude Shannon, for the first time in 1949 [43]. It is also known as Shannon entropy of randomness which is used to find the quality of encryption in the proposed system. The ideal value is always equal to 8 for which the pixel values of the image always fall in the range of 0–255. The entropy value may fluctuate for the various pixel values of the image falls. Hence the suggested cryptosystem has 256 states so the maximum information entropy will be approached to 8. The information entropy ‘m’ can be estimated by utilising the formula as

(12) $$H(m)=\sum_{i=0}^{2^{K}-1}p(m_{i})\log_{b}(1/p(m_{i}))$$

whereas $p(m_{i})$ is the probability of the message ‘m’, the $2^{K}$ in the above equation is the number of possible outcomes for the number of bits ‘K’ included for each message. The entropy values are evaluated for different standard images having a size of 512 × 512 × 3 and channels wise having a size of 512 × 512. The value must be nearer to the ideal value of 8. 

In Table 9 layer-wise entropy tests are applied for each encrypted image. The test is applied to eight different images. The topmost two images of splash and pepper are considered as the proposed standard images whilst the test is applied for remaining images as well. The values of 7.9992, 7.9991, 7.9993 for red, green, and blue channels of the proposed splash image is almost equal to 8. The test is further applied to the proposed standard test image of peppers having entropy values of red, green, and blue is 7.9993. The rest of the results are remarkable as shown below.

In Table 10 the combined values are calculated for eight standard images. The values of 7.9997 and 7.9998 reveal that the system has much randomness. In Table 11 the proposed system is compared to several eight types of already existing entropy values. The highly random values are difficult to be breakdown against any attack.

### 6.8. Time Complexity 

It is important to propose an efficient system. The system without efficiency has no value. The proposed system must be computationally fast and execution time takes fewer seconds to encrypt the channels and then for the full image as well. The proposed algorithm is tested on six test images and the execution time is noted for when the system encrypts the 512 × 512 channel and then encrypts the whole channel of 512 × 512 × 3. The time complexity test is done on the core i5 system having AMD Radeon graphics with 8 Gb ram. The proposed system in Table 12 shows that the system is much efficient. The unique system always takes the same time for encryption and decryption. The proposed cryptosystem is validated by the time complexity test. 

## 7. Software and System Specification 

The tests are performed for several test images having the size of 512 × 512 × 3 using the MATLAB 2017(a) version and workstation of ASUS CPU Core $i5^{TM}$(fourth generation) 8gb ram, AMD Radeon Graphics. The OS of the workstation is Windows 10. 

## 8. Conclusions

The paper proposed a hybrid chaotic fractal system consisting of fractal function and chaos-based three-dimensional chaotic map. The standard image was shuffled channel-wise prior to encryption. The encryption phase scrambled layer-wise images based on the multiplication operation using the Julia set of fractals. Furthermore, the encrypted layers are passed through the bitstream of the 3D Lorenz chaotic dynamical map for achieving higher security. The results of the proposed scheme were compared with existing secure algorithms. The addition of confusion and diffusion steps enhanced the robustness of the system when compared with traditional algorithms. The experimental analysis demonstrates that the suggested system indicated high sensitivity to initial conditions, strange attractor, aperiodicity, low correlation coefficient, high mean square error, and low peak to signal-noise ratio. The NPCR and UACI tests show that the proposed system is highly sensitive to a slight change in the plain image. The above security parameters have verified the proposed system for real-time communication. The proposed cryptosystem will be modified and tested for audio and video communication in the future. 

## Figures and Tables

**Table 1:** Adjacent pixel correlation analysis test for different test images.

		Correlation Coefficient Directions					
		Plain Image Directions			Ciphered Image Directions		
Image	Size	HD	DD	VD	HD	DD	VD
Splash	512 × 512	0.9839	0.9773	0.9913	0.0011	0.0037	0.0029
Pepper	512 × 512	0.9768	0.9639	0.9792	−0.0009	0.0033	0.0008
Tiffany	512 × 512	0.9381	0.8943	0.9409	0.0002	−0.0006	0.0057
Airplane	512 × 512	0.9663	0.9370	0.9641	0.0006	−0.0011	0.0029
Baboon	512 × 512	0.8665	0.7262	0.7587	−0.0038	0.0003	0.0007
Fruit	512 × 512	0.9738	0.9552	0.9743	0.0020	0.0022	0.0041

HD = Horizontal pixels direction, DD = Diagonal pixels direction, VD = Vertical pixels direction.

**Table 2:** Comparison of adjacent pixels with existing cryptosystems.

	Dimension	Correlation Coefficient Directions		
		HC	DC	VC
Plain image	512 × 512	0.9839	0.9773	0.9913
Proposed	512 × 512	0.0011	0.0037	0.0029
Ref. [67]	512 × 512	0.0075	0.0012	0.0049
Ref. [68]	512 × 512	0.0005	0.0008	0.0011
Ref. [69]	512 × 512	0.0117	0.0026	0.0010
Ref. [70]	512 × 512	0.0043	0.0054	0.0072
Ref. [71]	512 × 512	0.0108	0.0181	0.0061
Ref. [72]	512 × 512	0.0032	0.0042	0.0018
Ref. [73]	512 × 512	0.0204	−0.0174	0.0231
Ref. [74]	512 × 512	0.0053	−0.0027	0.0016

HC = Horizontal correlation, DC = Diagonal correlation, VC = Vertical correlation.

**Table 3:** Mean absolute error (MAE) values and its comparison.

Image	Dimension	MAE value	Ref. [75]	Ref. [56]
Splash	512 × 512	76	-	76
Pepper	512 × 512	79	74	74
Baboon	512 × 512	175	-	-
Tiffany	512 × 512	183	76	94
Airplane	512 × 512	125	74	-
Fruit	512 × 512	211	-	-

**Table 4:** Layer wise NPCR and UACI values of standard images.

			Projected Technique	
Image	Channels	Dimension	NPCR	UACI
Splash	R-L	512 × 512	99.62	33.90
	G-L	512 × 512	99.61	33.90
	B-L	512 × 512	99.62	33.90
Tiffany	R-L	512 × 512	99.61	36.26
	G-L	512 × 512	99.61	36.26
	B-L	512 × 512	99.60	36.26
Airplane	R-L	512 × 512	99.62	32.03
	G-L	512 × 512	99.61	33.05
	B-L	512 × 512	99.62	32.66

**Table 5:** Comparison of NPCR and UACI proposed cryptosystem and its comparison.

	Average NPCR	Average UACI
Proposed algorithm	99.62	33.90
Ref. [76]	99.52	26.79
Ref. [77]	99.58	33.37
Ref. [78]	99.59	17.60
Ref. [79]	99.60	33.23
Ref. [80]	99.60	28.13
Ref. [81]	99.55	33.40
Ref. [82]	99.59	33.46

**Table 6:** MSE and PSNR values of different layers for different standard images.

Image	Dimension		Projected Technique	
			MSE	PSNR
Splash	512 × 512	R-L	11412.96	7.59
	512 × 512	G-L	12272.97	7.28
	512 × 512	B-L	9908.84	8.20
Pepper	512 × 512	R-L	10926.92	7.78
	512 × 512	G-L	10809.52	7.83
	512 × 512	B-L	10790.86	7.83
Baboon	512 × 512	R-L	10917.79	7.78
	512 × 512	G-L	10889.19	7.79
	512 × 512	B-L	10909.10	7.79
Tiffany	512 × 512	R-L	17689.78	5.69
	512 × 512	G-L	13128.56	6.98
	512 × 512	B-L	07411.03	9.47
Fruit	512 × 512	R-L	11119.78	7.70
	512 × 512	G-L	09904.70	8.21
	512 × 512	B-L	09077.72	8.59
Airplane	512 × 512	R-L	09969.32	8.18
	512 × 512	G-L	10663.63	7.89
	512 × 512	B-L	10410.19	7.99

**Table 7:** Comparison of existing MSE values with proposed cryptosystem.

Algorithms	MSE values comparisons
AES	4600
AES-CBC	4637
AES-Counter	4938
AES Feedback	4577
AES-Stream	4911
Proposed	11198

**Table 8:** Average mean square error (MSE) and peak to signal noise ratio (PSNR) value.

	Projected Technique	
	Average MSE	Average PSNR
Splash	11198.25	7.69
Pepper	10842.43	7.81
Baboon	10905.36	7.79
Tiffany	12743.12	7.38
Fruit	10034.06	8.16
Airplane	10347.71	8.02

**Table 9:** Information entropy analysis for each respective channel.

		Encrypted Channels		
Images	Dimension	Red Channel	Green Channel	Blue Channel
Proposed 1	512 × 512	7.9992	7.9991	7.9993
Proposed 2	512 × 512	7.9993	7.9993	7.9993
Baboon	512 × 512	7.9993	7.9993	7.9992
Tiffany	512 × 512	7.9993	7.9994	7.9993
Airplane	512 × 512	7.9994	7.9994	7.9994
Fruit	512 × 512	7.9993	7.9993	7.9993
Pepper	512 × 512	7.9993	7.9993	7.9993
Lena	512 × 512	7.9994	7.9993	7.9994

**Table 10:** Cumulative entropy values for certain test images.

Test Images	Dimension	C-M Entropy
Ideal value	512 × 512 × 3	8.0000
Proposed 1	512 × 512 × 3	7.9997
Proposed 2	512 × 512 × 3	7.9998
Baboon	512 × 512 × 3	7.9997
Tiffany	512 × 512 × 3	7.9998
Airplane	512 × 512 × 3	7.9998
Fruit	512 × 512 × 3	7.9998
Pepper	512 × 512 × 3	7.9998
Lena	512 × 512 × 3	7.9998

C-M = Commulative measured.

**Table 11:** Comparison of proposed and Lena value with certain existing entropy values.

Images	Dimension	Entropy Values
Proposed	512 × 512 × 3	7.9997
Lena	512 × 512 × 3	7.9998
Ref. [83]	512 × 512 × 3	7.996
Ref. [84]	512 × 512 × 3	7.997
Ref. [85]	512 × 512 × 3	7.989
Ref. [86]	512 × 512 × 3	7.997
Ref. [87]	512 × 512 × 3	7.997
Ref. [88]	512 × 512 × 3	7.993
Ref. [89]	512 × 512 × 3	7.998
Ref. [22]	512 × 512 × 3	7.997

**Table 12:** Time complexity analysis for certain images.

Images	Dimension	Proposed Schemes	Ref. [53]	Ref. [90]
Splash	512 × 512	1.291540	-	-
Pepper	512 × 512	0.636624	2.76	3.68
Tiffany	512 × 512	1.302140	-	-
Airplane	512 × 512	1.059419	-	-
Fruit	512 × 512	1.098649	-	-
Baboon	512 × 512	0.609787	2.55	3.53
